# Composition and antimicrobial activity of *Rosmarinus officinalis* L. and *Artemisia monosperma* L. leaf essential oils and methanolic extracts from plants grown in normal and saline habitats in Egypt

**DOI:** 10.1038/s41598-024-57301-w

**Published:** 2024-03-28

**Authors:** Marwa Mohamed Soliman, Yasmin Mohamed Elsaba, M. S. A. Soliman, Eman Zakaria Ahmed

**Affiliations:** 1 Agricultural Research Center, Botanical Gardens Department, Horticulture Research Institute, Giza, Egypt; 2 Botany and Microbiology Department, Faculty of Science, Helwan University, Cairo, Egypt; 3 Nanotechnology Center, Helwan University, Cairo, 11722 Helwan Al Sharqia Egypt

**Keywords:** *Rosmarinus officinalis*, *Artemisia monosperma*, Essential oil, Salinity, Antioxidants, Antimicrobial activity, Biochemistry, Ecology, Microbiology, Physiology, Environmental sciences

## Abstract

The present work aimed to investigate the effect of salinity in natural habitats in Egypt on the main secondary metabolites of *Rosmarinus officinalis* L. and *Artemisia monosperma* L. plants compared to plants grown at normal conditions. Plants grown under salinity were collected from Egyptian Western Coastal region habitats irrigated with underground water. Results showed that salinity increased the essential oil percentage of *R. officinalis* L. by 52.7% and *A. monosperma* L by 0.29% in addition to the total phenolics and flavonoids content in dry leaves compared to control plants. GC/MS analysis of rosemary essential oils revealed that salinity decreased the amount of some major oil monoterpenes component as verbenone, with a slight effect on 1,8 cineole and increased Camphor, endo- Boreneol, and linalool in addition to the appearance of new specific components such as Chrysanthenone monoterpene ketone and Caryophyllene sesquiterpene, while, in the case of *Artemisia,* the GC/MS showed that Artemisia ketone, Camphor, β -phellandrene monoterpenes andα-Bisabolol sesquiterpenewere the major oil components; salinity decreased Camphor and β -phellandrene content and increased artemisia ketone and α-Bisabolol oil content. About 11 new oil constituents were detected such as ( +)-2-Bornanone and Sesquisabinene hydrate. Mineral ions (N, K^+^, Ca^+2^, P, and Mg^+2^) uptake by *R. officinalis* and *A. monosperma* decreased in plants grown under salinity, while Na content increased compared to corresponding controls. Results demonstrated that both plants could tolerate the high salinity level in natural Western Coastal region soil which promoted more production of valuable secondary metabolites. The antimicrobial effect of *R. officinalis* L. and *A. monosperma* L. leaf methanolic extracts, results showed that *R. officinalis* extracts had an inhibitory response against all tested gram-positive and negative bacteria, in addition to the yeast (*Candida albicans*), whereas there was no any inhibitory effect concerning *A. monosperma* L extract on the tested species.

## Introduction

Plant growth and development are adversely affected by many environmental stresses such as salinity, drought, flooding, heat, oxidative stress, and heavy metal toxicity. However, salinity stress is one of the major factors limiting agricultural production. According to recent reports, 20% of land worldwide is subjected to salinity stress^1^. In recent decades, the increase in salinization of soils and ground waters is considered to be a major problem of agriculture in Egypt and is thought to be a result of the Nile’s weak demineralization of the soil. It is also facilitated by the absorption and accumulation of salts in quantities that are toxic to plants^2^. Soil salinization suppresses the growth of many economic agricultural plants. Moreover, the availability of non-saline water for irrigation is limited, and its quality continues to decline in arid and semi-arid areas. Therefore, saline water usage in agriculture currently seems to be an urgent solution. Cultivation of resistant medicinal and aromatic plants is an option to utilize these soils. Rosemary and *Artemisia* are wild plant species originating in the Mediterranean region; they constitute an interesting solution to avoid desertification and rapid soil erosion due to their high tolerance to environmental stresses such as salinity.

An important member of the Lamiaceae family, *R. officinalis* L., is native to the Mediterranean Sea coasts of Egypt. *R. officinalis*, are cultivated as a medicinal plantin different regions of the world, such as the Mediterranean, Asia, and Latin America^3^. It is characterized by high content of aromatic phenolic compounds with nutraceutical and pharmaceutical properties, including ant obesity, anti-inflammatory, antidiabetic, diuretic, antithrombotic, antimicrobial, anticancer, hepatoprotective, and antioxidant^4^. Numerous studies have highlighted that the majority of these biological activities are correlated with the phenolic composition^5^. Its essential oil is utilized also for a variety of purposes, including aromatherapy^6,7^, pest control products, and flavoring and fragrance^8,9^. 1,8-cineole, camphor, -pinene, -pinene, and borneol are the active components of rosemary essential oils^10^.

*Artemisia monosperma* L. is a member of the Asteraceae family. In Chinese herbal medicine (CHM), it is also referred to as Sweet Annie, wormwood, or Qing Hao, and it is used for a variety of ailments including fever and malaria. *A. monosperma* has been found to have significant bioactive components like artemisinin, endoperoxide sesquiterpene lactone, and essential oil (EO)^11^. Several *Artemisia* species grow wildly or as cultivated plants for their use as medication and as a herbal tea preparation in the Mediterranean region^12^. The leaves of *Artemisia* have been shown in prior studies to possess some biological activities, including antifungal, antimicrobial, antimalarial, antibacterial, anti-inflammatory, anti-tumor, and antiallergenic qualities^13,14^.

Aromatic plants respond to stress conditions through different physiological defense mechanisms by secondary metabolites production, which are toxic to insects, micro-organisms and/or herbivore repellent. Essential oil percentage and composition are affected by a range of environmental factors including climate, pollution, and exposure to pests or diseases^15^. The stress factor resulted in different changes in the essential oil (EO) composition of some aromatic plants such as rosemary, Sweet Annie, mint, oregano, and basil^16^.

Although salinity is considered an abiotic stress with a negative effect on most plants, it can also function as a promotive and driving force on plant secondary metabolites. The objective of the current study is to investigate the effect of salinity on growth and some secondary metabolites content of two important salt-tolerant medicinal plants *R. officinalis* L. and *A. monosperma *L.with high economical valuable secondary metabolites.

## Materials and methods

*R. officinalis* L. and *A. monosperma* L. plants grown under salinity were collected from Egyptian Western Coastal region 10m Elevation (30 ͦ 51′ 5″ N, 29 ͦ 18′ 25″ E) in which underground water was used for irrigation and the control specimens were collected from commercial farm in Cairo where Nile water was used for irrigation. Soil samples were collected from the two habitats for analysis (Table1).

**Table 1:** Soil and water chemical analysis in ppm.

Soil and water samples	Mg	K	Ca	Na	P
	ppm				
Salinity soil	0.077	0.087	0.233	1.07	0.011
Control soil	0.090	0.066	0.195	0.23	0.010
Salinity water	177.15		117.64	22.35	0.05
Control water	10.80		30.36	0.91	0.03

For secondary metabolites detection, aerial parts of *R. officinalis* L. and *Artemisia monosperma* L. plants were collected, air dried to complete dryness.

Leaves essential oil was extracted using hydrodistillator as traditional method for extraction of bioactive compounds, mainly essential oils from plants^17^. Mohammed et al.^18^, revealed that the best volatile oil yield by the hydrodistillation procedure of oil extractions. Hydro distillation was carried out for 2h and repeated with total 3 replicates for each. Finally, the essential oils were stored at 4 °C for GC/MS analysis, which was carried out at the Agriculture Research Centre, Cairo, Egypt, as follows.

### Gas chromatography–mass spectrometry analysis

(GC–MS) system (Agilent Technologies) was performed using gas chromatograph (7890B) and mass spectrometer detector (5977A) Samples were diluted with hexane (1:19, v/v). The GC was equipped with HP-5MS column (30 m × 0.25 mm internal diameter and 0.25 μm film thickness). Analyses were carried out using hydrogen as the carrier gas at a flow rate of 1.0 ml/min at a split 1:20 of, injection volume of 1 µl and the following temperature program: 40 °C for 1 min; rising at 4 °C /min to 150 °C and held for 6 min; rising at 4 °C/min to 210 °C and held for 1 min. The injector and detector were held at 280 °C and 220 °C, respectively. Mass spectra were obtained by electron ionization (EI) at 70 eV; using a spectral range of m/z 50–550 and solvent delay 4 min. Identification of different constituents was determined by comparing the spectrum fragmentation pattern with those stored in Wiley and NIST Mass Spectral Library data.

*Total phenolics and flavonoids estimation* were extracted by adding 0.1 g powdered air-dried leaves to 25 ml methanol 80% at 60 °C for 2 days with continuous stirring. After filtration the extract was used for the estimation of total phenolics and flavonoids.

*Total phenolic* content was determined according to Kujala et al.^19^, using Folin–Ciocalteu reagent and gallic acid as a standard. Briefly, 0.5 ml of filtered extract was added to 2.5 mL Folin-Ciocalteu’s reagent (diluted with ethanol 1:10), 2 mL of Na_2_CO_3_ (7.5%) and mixed well. After 15 min incubation at room temperature, the absorbance of mixtures was recorded by Jenway 6405 UV–Vis spectrophotometer at 765 nm. The total phenolic content was expressed as mg gallic acid equivalent (GAE) per gram of extract (mg GAE/g dry weight of extract).

*Total flavonoids* content was determined according to Zhishen et al.^20^ and Paciolla et al.^21^, where 0.5 ml of the extract was added to 150 µl of 5% sodium nitrate and allowed to stand for 6 min. Then 150 µl of 10% Aluminum chloride solution was added and allowed to stand for 6 min after which 200 µl of 1 M sodium hydroxide was added then the mixture was completed to 5 ml with methanol and mixed well. After incubation for 15 min, the absorbance was measured spectrophotometrically against a blank at 510 nm. The total flavonoids content was expressed in milligrams of quercetin equivalents (QE) per gram extract (mg QE/g).

### Antimicrobial activity

Methanolic extracts were prepared from air dried leaves, 4g powdered leaves were extracted by 200 ml methanol 80% at 60 °C for 2 days with continuous stirring then the extracts were allowed to dry at 40 °C until complete dryness and redissolved w/v in methanol for preparation of known concentrations.

#### Tested microorganisms

Antibacterial activity was estimated in vitro against six pathogenic bacterial strains including *Staphylococcus aureus* (ATCC 6538), *Clostridium perfringens* (ATCC 13,124) and *Micrococcus leutus*, as Gram positive stains while *Escherichia coli* (ATCC 5739), *Shigella sonnei* (ATCC 29,930) and *Salmonella typhimurium* (ATCC 14,028) as Gram negative stains. In addition, the pathogenic yeast *Candida albicans* was used.

#### Well diffusion assay

*R. officinalis L. and A. ****monosperma***’s antibacterial activity was in vitro investigated using the agar well diffusion method^22^. 100 µl of 24 h old microbial culture of the tested strains with a concentration equivalent to 0.5 McFarland (1.5 × 10^8^ CFU ml^–1^) were streaked on the surface of the agar plates (nutrient agar media for bacteria and potato dextrose agar media for yeast). Further, under aseptic conditions, 100 µl of either *R. officinalis L.* or *A. ****monosperma*** extracts were inoculated into the 0.8 cm wells at concentration 25 mg ml^-1^ in methanol. Clindamycin (2.0 mg/disk), chloramphenicol (30 µg/disk) and nystatin (100,000 IU/ml) were used as positive controls, while methanol was used as a negative control. The incubation was carried out at 35–37 °C for 24–48 h to allow the growth of bacteria and yeast. The inhibition zone formation around the wells were recorded and measured in mm. The experiment was carried out in triplicate and the means of inhibition zones were measured in millimeters ± standard deviation.

## Results

**Figure 1:**
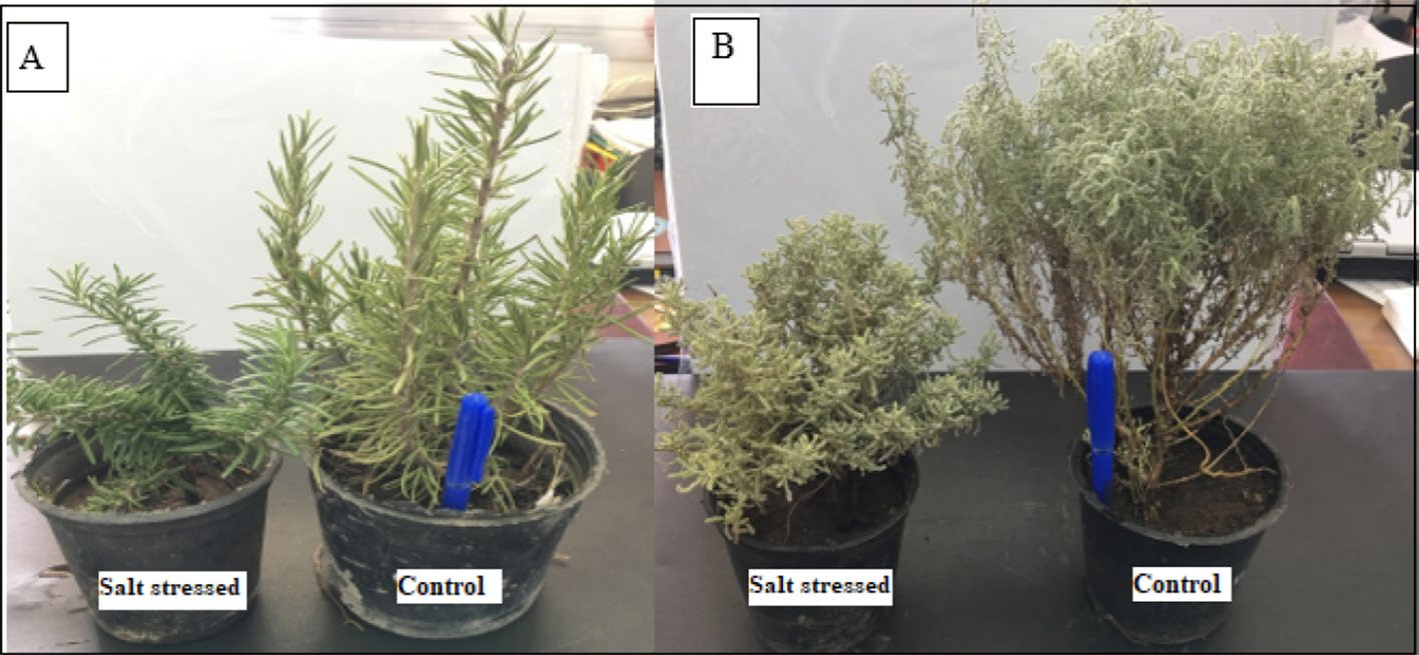
Photos for *R. officinalis* L. (**A**) and of *A.monosperma* L. (**B**) in salty soil and control ones.

In the present study, a great decrease in shoot vegetative growth of *R. officinalis* and *A. monosperma* grown under salinity in the Egyptian Western Coastal region was noticed (Fig. 1). Data illustrated in Table 2 shows that under salinity stress, the uptake of N, Mg^2+^, Ca^2+^, K^+^, and P decreased while Na^+^ ion concentration increased in both *R. officinalis* L. and *A.monosperma* L. plants significantly compared to corresponding controls. Plant shoot Na^+^ concentration rises as salinity rises, while shoot K^+^ and the K^+^/Na^+^ ratio decreases.

**Table 2:** Mineral ions content of *R. officinalis* and *A. monosperma* air dry leaves.

Mineral ions in ppm	N	P	Mg	K	Ca	Na	Mg
Control rosemary	3.64	0.16	1.3	4.5	8.1	1	1.3
Salinity rosemary	2.23	0.12	1.2	3.9	7.9	3.5	1.2
Control artemisia	4.31	0.19	1.9	8.6	11.05	6.6	1.9
Salinity artemisia	2.29	0.15	1.6	6.3	2.8	13.1	1.6

**Figure 2:**
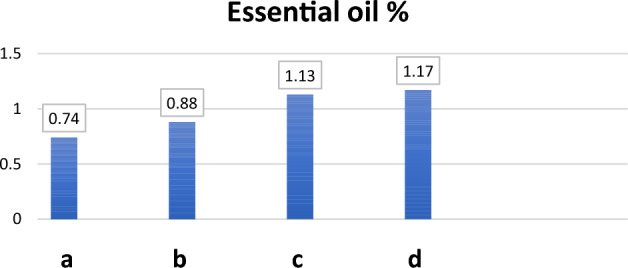
Effect of salinity on essential oils percent in *R. officinalis* and *A.monosperma* plants (a & c represents Control plants while, b & d represents Salt stressed plants).

Salinity increased essential oil (EO) percent on a dry weight basis of *R. officinalis* and *A. monosperma* plants more than their relative controls (Fig. 2). The study of mass spectra identified 15 compounds in the essential oils of control rosemary plants while 18 compounds were detected in salt-stressed plants. According to GC/MS analysis of essential oils from dry aerial parts of *R. officinalis,* the main constituents of *R. officinalis* oil were found to be Verbenone, α-pinene, 1,8cineole, and Camphor in control plants, whereas in the essential oil of salt-stressed plants, Verbenone, Camphor, endo-Borneol and 1,8cineole were the main components. Salinity decreased Verbenone from 39.48 to 26.15%, α-pinene from 15.52 to 4.16%, with slight effect on 1,8cineole while increased Camphor, endo- Boreneol and linalool from 8.57%, 4.39%, 2.12% to 22.2%, 16.8% and 6.31% respectively in addition to the appearance of new specific components which were Chrysanthenone and Caryophyllene under salinity stress (see Table 3). Simultaneously, the analysis of *Artemisia monosperma* L essential oil showed that ketone, Camphor, α-Bisabolol, and β -phellandrene were the main oil components. Salinity decreased Camphor and β -phellandrene content yet increased ketone and α-Bisabolol content in *Artemisia* oil at the same time. About 11 new oil constituents were detected such as ( +)-2-Bornanone and Sesquisabinene hydrate (see Table 3). In comparison to rosemary, *A. monosperma* plants often contain more phenolics and flavonoids (see Table 4). Salinity increased the total leaves content of phenolics (288.63 and 85.93 mg g^–1^) and flavonoids (61.6 and 10.5 mg g^–1^) in both *R. officinalis* L. and *A. monosperma* L. plants, respectively, compared to their corresponding controls phenolics (206.25 and 66.60 mg g^–1^) and flavonoids (40.5 and 9.23 mg g^–1^).

**Table 3:** GC-Mass analysis of *R. officinalis* and *A. monosperma* essential oils in both control and salt affected plants.

RT	Compound name		Control %			Salt %
(a) GC/MS analysis of *R. officinalis* L. essential oil						
15.33	Verbenone	Monoterpene	39.48		26.1	
4.94	α-pinene	Monoterpene	17.52		4.16	
8.40	1,8cineole	Monoterpene	10.17		10	
12.77	Camphor	Monoterpenes	8.57		22.2	
14.76	α-Terpineol	Monoterpenes	5		1.81	
18.08	Bornyl acetate	Monoterpene	4.74		0.77	
12.96	cis-Verbenol	Monoterpene	1.23		4.76	
13.70	endo-Borneol	Monoterpene	4.39		16.8	
14.20	Terpinen-4-ol	Monoterpene	2.36		0.93	
11.34	Linalool	Monoterpene	2.12		6.31	
5.41	Camphene	Monoterpene	1.3		0.87	
16.56	Shisool	Monoterpene	1.21		2.04	
13.93	Bicyclo[3.1.1]heptan-3-one, 2,6,6-trimethyl-, (1.alpha.,2.beta.,5.alpha.)-		1.15		1.97	
16.37	CIS-D-Dihydrocarveol	Monoterpene	0.74		–	
12.12	Chrysanthenone	Monoterpene ketone	–		0.68	
22.44	Caryophyllene	Sesquiterpenoid	–		0.57	
(b) GC/MS analysis of *A. monosperma* L. essential oil						
Artemisia						
9.79	Artemisia ketone	Monoterpene		31.66	41.04	
16.88	Camphor	Monoterpene		16.88	–	
30.93	.alpha.-Bisabolol	Sesquiterpene		11.62	21.28	
8.63	.beta.-Phellandrene	Monoterpene		10.21	1.55	
6.56	Sabinene	Monoterpene		4.81	0.74	
7.25	.beta.-Myrcene	Monoterpene		2.44	0.19	
6.62	2-.BETA.-PINENE	Monoterpene		2.28	–	
36.27	1,6-Dioxaspiro[4.4]nona-2,8-diene, 7-(2,4-hexadiynylidene)-			2.09	1.23	
28.92	Isospathulenol	Monoterpene		1.48	0.46	
29.64	Bisabolol oxide	Sesquiterpene		1.46	0.98	
5.64	Camphene	Monoterpene		1.43	0.37	
35.11	3-(1,5-Dimethyl-hex-4-enyl)-2,2-dimethyl-cyclopent-3-enol			1.3	–	
35.53	3,7-Cycloundecadien-1-ol, 1,5,5,8-tetramethyl-			1.11	–	
13.84	Isoborneol	Monoterpene		1.01	1.5	
14.62	Cryptone	Monoterpene		1	–	
27.45	Caryophyllene oxide	Monoterpene		0.97	0.46	
14.29	Terpinen-4-ol	Monoterpene		0.86	-	
7.63	3,6-Heptadien-2-ol, 2,5,5-trimethyl-, (E)-			0.82	1.58	
10.65	Artemisia alcohol	Monoterpene		0.76	1.55	
14.84	(1R)-(-)-Myrtenal	Monoterpene		0.71	0.34	
4.43	Santolina triene	Monoterpene		0.58	0.13	
27.08	Nerolidol	Sesquiterpene		0.52	0.23	
27.36	(-)-Spathulenol	Sesquiterpene		0.52	–	
29.08	10,10-Dimethyl-2,6-dimethylenebicyclo [7.2.0]undecan-5.beta.-ol			0.44	0.14	
29.26	Humulenol-II			0.44	0.22	
28.25	Limonen-6-ol, pivalate	Monoterpenes		0.41	–	
24.58	Benzene, 1-(1,5-dimethyl-4-hexenyl)-4-methyl			0.4	–	
4.97	.Alpha.-pinene, (-)-	Monoterpene		0.39	0.08	
21.67	.Beta. Elemene	Sesquiterpene		0.38	0.14	
14.99	Myrtenol	Monoterpene		0.34	0.22	
33.25	7-Isopropenyl-1,4a-dimethyl-4,4a,5,6,7,8-hexahydro-3H-naphthalen-2-one	Sesquiterpenes		0.33	–	
24.41	Germacrene-D	Sesquiterpene		0.32	0.69	
12.81	( +)-2-Bornanone	Monoterpene		–	22.82	
27.27	Sesquisabinene hydrate	Sesquiterpene		–	1.22	
27.69	Cis-Sabinene hydrate	Monoterpene		–	0.08	
36.98	1,6-Octadiene, 5,7-dimethyl-, (R)-	Monoterpene		–	0.21	
13.55	Pinocarvone	Monoterpene		–	0.16	
28.26	Chrysantenyl 2-methuylbutanoate	Monoterpene		–	0.15	
24.14	cis-Verbenol	Monoterpene alcohol		–	0.05	
22.20	1,5-Cyclooctadiene, 1-t-butyl-			–	0.08	
25.49	(Z,E)-.alpha.-farnesene	Sesquiterpene		–	0.07	
22.45	trans-.alpha.-Bergamotene	Sesquiterpene		–	0.06

**Table 4:** Total phenolics and flavonoid content of *R. officinalis* and *A. monosperma* L. air dry leaves.

Treatment	Phenolics	Flavonoids
	mg g^–1^ dry leaves	
*A.monosperma* (salt stressed)	288.63	61.6
*A.monosperma* (control)	206.25	40.5
Rosemary (salt stressed)	85.93	10.5
Rosemary (control)	66.60	9.23

**Figure 3:**
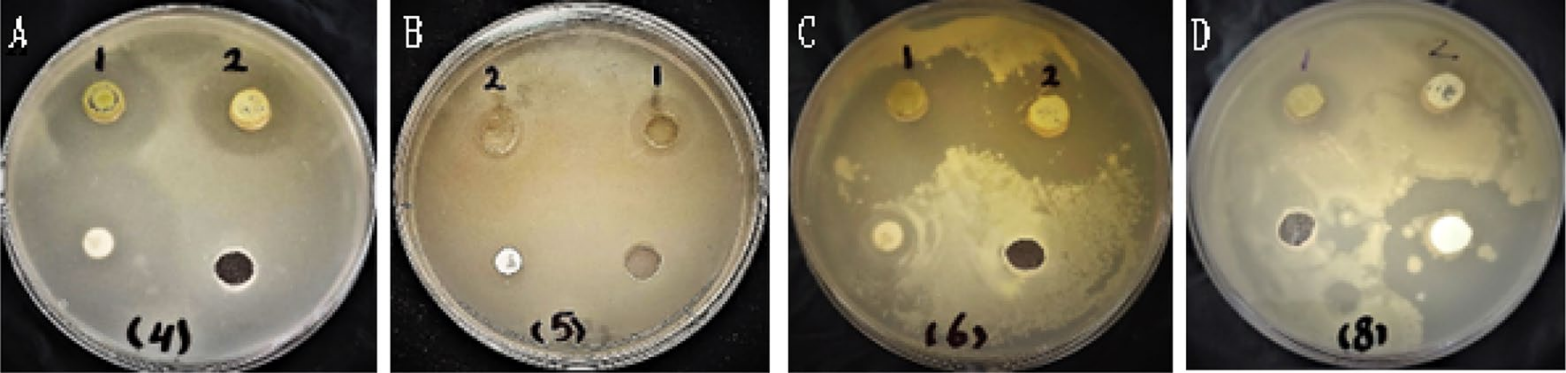
Antibacterial activity of *Rosmarinus officinalis* methanolic extracts. 1, is the treated extract, 2 is the control extract. (**A**) *Staphylococcus aureus*, (**B**) *Clostridium perfringens* (**C**) *Micrococcus luteus*, (**D**)* Candida albicans.*

The findings of the antimicrobial assay conducted on *R. officinalis* L. and *A. monosperma* L revealed that only the methanolic extracts of *R. officinalis* L. exhibited inhibitory effects against the tested microorganisms specially the Gram-positive bacteria and the pathogenic yeast. The highest activity was observed against *Micrococcus luteus* followed by *Staphylococcus aureus*, and *Clostridium perfringens*, with recorded inhibition zones of 37 mm, 30 mm, and 16 mm, respectively, using the treated *R. officinalis* L.extract. These values were higher than those recorded for the control extract, which exhibited inhibition zones of 35 mm, 24 mm, and 13 mm respectively. On the other hand, resistance response was shown by all the tested Gram-negative bacteria. Furthermore, the antimicrobial assay revealed that the treated *R. officinalis* L extract exhibited a stronger inhibitory response against the pathogenic yeast *Candida albicans* compared to the control extract with inhibition zones of 18 mm and 14 mm, respectively (see Table 5 & Fig. 3).

**Table 5:** Antimicrobial activity of the methanolic extracts of *Rosmarinus officinalis* and *Artemisia monosperma* L. plants.

Tested microorganisms	Rosemary		Artemisia		– ve control (methanol)	+ ve control		
	Treated	Control	Treated	Control		Clindamycin	Chloramphenicol	Nystatin
*Staphylococcus aureus*	30	24	0	0	0	42	Nd	Nd
*Clostridium perfringens*	16	13	0	0	0	4	Nd	Nd
*Micrococcus leutus*	37	35	0	0	0	10	Nd	Nd
*Escherichia coil*	0	0	0	0	0	0	10	Nd
*Shigella sonnei*	0	0	0	0	0	Nd	0	Nd
*Salmonella typhimurium*	0	0	0	0	0	Nd	10	Nd
*Candida albicans*	18	14	0	0	0	Nd	Nd	31

Inhibition zone in millimeter.

*Nd* not detected.

## Discussion

Arid and semi-arid regions face a high accumulation of salt in soil and underground water. Salinity stress is one of the abiotic stresses that harm agriculture by decreasing plant vegetative growth and productivity. Salinity hampers photosynthetic machinery, transpiration, and gaseous exchange by decreasing the content of chlorophyll and carotenoids, distorting chloroplast ultrastructure and PSII system, and reducing stomatal conductance^23^.

Salinity leads to an extensive accumulation of ions (Na^+^ and Cl^−^) and inhibits K^+^ and Ca^2+^ uptake resulting in ionic imbalance^24^ which enhances reactive oxygen species (ROS) production in plant cells and creates oxidative stress causing uncontrolled damage to cell macromolecules including membranes lipid peroxidation DNA, and protein damage^25^. By upsetting the equilibrium of nutrient intake that the plant system maintains, salinity directly affects plant growth. The most significantly impacted are nutrient availability, partitioning, and transport due to the competition between the nutritional ions K^+^, Ca^2+^, and NO_3_^-^ and the Na^+^ and Cl^-^ions. The excess of Na^+^ and Cl^–^ ions directly causes these ionic imbalances impacting the biophysical and/or metabolic elements of the plant system. Many plants have shown an increase in Na^+^ and Cl^–^ during salt stress, while fennel, *Trachyspermum ammi*, peppermint, *lemon verbena*, *Matricaria recutita*, and *Achillea fragratissima* showed a decline in N, P, K^+^, Ca^2+^, and Mg^2+^ levels^26–28^.

Ali and Hassan^29^ stated that N, P, Ca^2+^, and K^+^ percentages were influenced by different salt concentrations in the leaves of *Simmondsia chinensis* (jojoba). Salt concentration over 17.2 mM drastically lowered the mentioned elements' uptake and content in the plant. Plant responds to saline conditions in terms of various strategies and approaches such as ion homeostasis and compartmentalization, transport of ions, osmotic adaptation, stimulation of antioxidant machinery, and biosynthesis of secondary metabolites^30^.

Essential oil is one of the most valuable secondary metabolites in aromatic plants. The essential oil (%) extracted from chamomile, lemon verbena, and peppermint increased under salinity stress levels as reported by Ref.^31^. Gharib et al.^32^ also reported a 32.33% increase in the rosemary oil percentage under salinity stress (100 mM NaCl) in irrigation water. Stimulation of essential oil production under a moderate degree of salinity could be due to a higher oil gland density and an increase in the absolute number of glands produced prior to leaf emergence because of a stress-induced reduction in leaf area^33^ and secondary metabolites synthesis and accumulation as self-defense components to cope with stressful conditions^32^. The increase in the essential oil content in some of the salt-stressed plants might be also attributed to a decline in the primary metabolites, causing intermediary products to become available for secondary metabolite synthesis. Despite the bulky structures, caryophyllene easily penetrates cell membranes^34^, which determines their bioavailability and a variety of biological properties including antioxidant and anti-inflammatory properties. *R. officinalis* essential oil also exhibits free radical scavenging activity and excellent hepatoprotective properties by limiting the lipid peroxidation. The 1,8-cineole from *R. officinalis* essential oil is a lipid-soluble compound that facilitates passage across the blood brain barrier, producing neuronal effect on the receptor sites or impacting enzyme activity of neurons^35^. Mohammed et al.^18^, stated that the major rosemary oil constituents were 1,8-cineole, camphor, and camphene, these three major constituents is about 50.9% of the total oil compositions. As the time of harvesting also affects the oil content he also reported that the yields of camphene, β-pinene, α-terpineol, bornyl acetate, β-caryophyllene, and d-germacrene increased in the two- and three-weeks dried herbs-based volatile oils as compared to the fresh and one-week dried herbs-based oils. Sarmoum et al.^36^ reported that 1,8cineole content in rosemary essential oil decreased up to 50% with increasing NaCl concentrations (from 25 to 200 mM). This agrees with Langroudi et al.^37^ who recorded those different levels of salinity decreased 1,8-cineole, borneol, camphor, and α-pinene the main constituents of *R. officinalis* essential oils in Iran. Salinity decreased monoterpene hydrocarbons including very low α-pinene and camphene, whereas oxygenated monoterpenes recorded the highest value including high camphor and borneol yet resulted in lower values of 1,8-cineol as compared with control rosemary plants^32^. The phytochemical analysis of *Artemisia judaica* essential oil revealed the dominance of the highly active antioxidant volatile compounds, oxygenated monoterpenes, and cinnamic acid derivatives in the essential oil constituents of the plant. Such classes of compounds were reflected in the in vitro and in vivo potential antioxidant activity of essential oil^14^. *Artemisia monosperma* L. plants adapted to salinity produced higher values of EO (%) and EO constituents [α-pinene, camphene, coumarin, and dihydro-epi-deoxyarteannuin B^38,39^. To reduce damage, plants have evolved complex antioxidative systems, involving antioxidant enzymes and secondary metabolites like phenolic compounds^40^. Mehrizi et al.^41^ and Abd EL Azim et al.^27^ reported an increase in phenolics and flavonoids under salinity from 50 mM to below 100 mM in *R. officinalis* and *Achillea fragratissima*.

Free radicals can be effectively eliminated by flavonoids and other phenolics^42,43^ improving plant tolerance to salinity stress. Antioxidants inhibit the oxidation of lipids, proteins, and DNA; hence, tolerant plants tend to increase the production of phenolics under salinity stress. Phenols increased in *Achillea fragratissima* with increased salinity levels^27^ the same result was reported in *R. officinalis*^44^. Salinity usually causes a reduction in growth providing an additional carbon skeleton for phenols indicating that salinity is a more effective factor in increasing phenols i.e., the natural antioxidants. An increase in the total phenols content of tolerant genotypes could be an adaptive mechanism for preventing damage during stress^45^. The GC-Mass analysis results showed that α-Terpineol and Terpinen-4-ol, represented major constituents of the *R. officinalis* as similarly reported by Ref.^46^. These compounds are known for their antibacterial activity due to their lipophilic nature which causes the disruption of the lipopolysaccharide in the bacterial membrane, causing cell disruption^47^. Interestingly, the need for new antimicrobial agents has become increasingly crucial due to the rise of microbial resistance. Fortunately, the present results showed the promising antimicrobial activity of the methanolic *R. officinalis* extract specially against *Micrococcus luteus*, *Staphylococcus aureus*, *Clostridium perfringens* and *Candida albicans*.

Manilal et al.^48^ investigated the antibacterial activity of *R. officinalis* extract against multidrug-resistant clinical isolates and meat-borne pathogens, and reported that *Salmonella* sp. and *S. aureus* were the most sensitive clinical isolates.

According to Gomez-Estaca et al.^49^, rosemary oil prevented the growth of typical food bacteria that cause food to spoil. Burt^50^ has demonstrated the rosemary essential oil's antibacterial efficacy against *S. aureus, Bacillus cereus,* and *E. coli*. Additionally, Sirocchi et al.'s research^51^ demonstrated that *Brochothrix thermosphacta* and *Enterobacteriaceae* growth was suppressed by rosemary essential oil. Moreover, Jafari-Sales & Hossein-Nezhad (2020) reported the antibacterial activity of the methanolic *R. officinalis* extract *against S. aureus, B. cereus, E. coli* and *Pseudomonas aeruginosa.* Also, Mattazi et al.^52^ reported the antibacterial activity of *R. officinalis* EO against *Micrococcus luteus*.

*R. officinalis* extract components act in synergy and interact with the bacterial cell membrane, affecting the generation of fatty acids, genetic material, and nutrients as well as the transfer of electrons, cellular component leakage, and fatty acid transport. Additionally, it caused a protein interaction with the membrane that resulted in the loss of membrane structure and functionality^53^.

The results also showed a promising antifungal activity of the rosemary extract against *Candida albicans.* This may be due to the presence of various compounds in *R. officinalis* extract which had antifungal properties such as 1,8 cineole and camphor as well as phenolics and flavonoids^54^. Similarly, Saeidi et al.^55^ reported the inhibitory effect of *R. officinalis* extract in concentration of 100 µg/mL on *Candida albicans.*According to Zaouali et al.’s findings, the effectiveness of *R. officinalis* bioactive compounds is related to the combined action of the various minor components present in its volatile and nonvolatile fractions and should not be associated with the action of any particular component^56^.

## Conclusion

In conclusion, aromatic plants are a safe and rich source of natural secondary metabolites with many reported biological activities. *R. officinalis* and *A. monosperma* can be planted in moderate saline soil where stressed plants tend to increase essential oil percentage and total phenolics content compensating for the decrease in growth. This may help in making use of many salt-affected arid and semiarid land areas. The results showed that rosemary methanolic extract could be a promising alternative antimicrobial source against various pathogenic bacteria and yeast, helping in drug resistance issues which is a global health problem. Further optimization studies could be performed to enhance the antimicrobial potency and for the best exploitation of *R. officinalis*.

## Data Availability

The datasets used and/or analysed during the current study are available from the corresponding author on reasonable request. Experimental research and field studies on plants, including the collection of plant material, comply with relevant institutional, national, and international guidelines and legislation. Identification of the plant material used in the study was performed by Prof. Dr. Mohamed Soliman and Prof. Dr. Loutfy Mohsen at Faculty of Science- Helwan University – Botany and Microbiology department. Some of collected samples was kept in Helwan University herbarium (vouchers no 00000897 and 00000999).
